# Upper extremity asymmetry due to nerve injuries or central neurologic conditions: a scoping review

**DOI:** 10.1186/s12984-023-01277-7

**Published:** 2023-11-09

**Authors:** Sandesh G. Bhat, Alexander Y. Shin, Kenton R. Kaufman

**Affiliations:** 1 Department of Orthopedic Surgery, Mayo Clinic, Rochester, MN 55905 USA; 2 Motion Analysis Laboratory, Mayo Clinic, DAHLC 4-214A, 200 First Street SW, Rochester, MN 55905 USA

**Keywords:** Upper extremity asymmetry, Neurological conditions, Peripheral nerve injury, Central neurological injuries, Scoping review

## Abstract

**Background:**

Peripheral nerve injuries and central neurologic conditions can result in extensive disabilities. In cases with unilateral impairment, assessing the asymmetry between the upper extremity has been used to assess outcomes of treatment and severity of injury. A wide variety of validated and novel tests and sensors have been utilized to determine the upper extremity asymmetry. The purpose of this article is to review the literature and define the current state of the art for describing upper extremity asymmetry in patients with peripheral nerve injuries or central neurologic conditions.

**Method:**

An electronic literature search of PubMed, Scopus, Web of Science, OVID was performed for publications between 2000 to 2022. Eligibility criteria were subjects with neurological conditions/injuries who were analyzed for dissimilarities in use between the upper extremities. Data related to study population, target condition/injury, types of tests performed, sensors used, real-world data collection, outcome measures of interest, and results of the study were extracted. Sackett’s Level of Evidence was used to judge the quality of the articles.

**Results:**

Of the 7281 unique articles, 112 articles met the inclusion criteria for the review. Eight target conditions/injuries were identified (Brachial Plexus Injury, Cerebral Palsy, Multiple Sclerosis, Parkinson’s Disease, Peripheral Nerve Injury, Spinal Cord Injury, Schizophrenia, and stroke). The tests performed were classified into thirteen categories based on the nature of the test and data collected. The general results related to upper extremity asymmetry were listed for all the reviewed articles. Stroke was the most studied condition, followed by cerebral palsy, with kinematics and strength measurement tests being the most frequently used tests. Studies with a level of evidence level II and III increased between 2000 and 2021. The use of real-world evidence-based data, and objective data collection tests also increased in the same period.

**Conclusion:**

Adequately powered randomized controlled trials should be used to study upper extremity asymmetry. Neurological conditions other than stroke should be studied further. Upper extremity asymmetry should be measured using objective outcome measures like motion tracking and activity monitoring in the patient’s daily living environment.

## Introduction

It is estimated that 795,000 people suffer a stroke in the United States yearly of which 70% are first time strokes [1]. Between 2015 and 2018, 7.6 million people suffered a stroke [2]. Cerebral Palsy (CP) affects more than 17 million people worldwide [3–6]. Each year, about 60,000 Americans are diagnosed with Parkinson’s disease (PD) [7]. In 2020, the number of known cases of Multiple Sclerosis (MS) increased to 2.8 million worldwide [8]. Amyotrophic Lateral Sclerosis (ALS) affected about 12,187 people in the United States between 2010 and 2011 [9].

Traumatic injuries resulting in peripheral nerve injury (PNI) (including brachial plexus injury (BPI)) and spinal cord injury (SCI) cause extensive disabilities in the upper extremity (UE). Motor vehicle accidents (MVA) are the predominant cause of PNI, where 5% of all MVAs result in a form of PNI [10]. About 8% of PNI patients have a BPI [11]. Adult traumatic BPI results in severe impairment following penetrating wounds, falls, and MVA or other high-energy trauma. Young male adults comprise a majority among patients with a BPI [12]. MVAs and falls are a leading cause of SCI. 38.1% of all SCI were caused by MVAs and 53% by falls between 2010 and 2014 [13]. The United States has an estimated annual SCI incidence of 17,000 [14]. The National Spinal Cord Injury Statistical Center estimated 282,000 people were living with a SCI in 2016 [15].

Conditions/injuries affecting the nervous system can be debilitating. Stroke victims suffer paretic limbs [16] and is the third-leading cause of disability [17]. CP accounts for most of the lifelong neurological disabilities [3–6]. Bimanual coordination impairments were found in children with CP [18–20]. BPI predominately affects young and otherwise healthy men resulting in paralytic upper extremities [21]. Patients with MS display reduced gross or fine motor capabilities, in addition to slowness, clumsiness, and dysmetria [22–24], while patients with PD displayed a deficit in inhibitory control [25–30]. Many of the UE PNI patients, despite rehabilitative efforts, never achieve satisfactory motor recovery [31–34]. All of these neurological conditions/injuries can affect the patient’s ability to perform daily tasks with their affected UE.

A wide range of outcome measures have been described in the literature as well as clinical practice to quantify the disabilities of the UE. These measures involve tests and surveys on dexterity, strength, pain, disability, amount of activity, etc. When the neurological condition/injury is unilateral, the differences in the outcome measures between the ipsilateral and contralateral sides can be used to gauge the patient’s recovery or the progression of the condition. Such a comparison between the affected and the unaffected sides is beneficial and provides patient specific, real-world evidence (RWE) [35] based data and information. Real world evidence is a combination of data collected outside of a traditional clinical setting. Asymmetry measures can either be subjective or objective, measured in a clinical setting or in the patient’s daily living environment. Clinical surveys to measure outcomes are either too extensive or too limited in scope, are completed without supervision and is time intensive for patients with a relatively high rate of failure to complete. This emphasizes the importance to understand the benefits and shortcomings of each type of test in relation to the treatment population.

Previous reviews have explored wearable systems [36–38], fine and gross motor tests [39], and various functional evaluation techniques [40] for UE assessment and rehabilitation. No currently available review or article explores the state of the art in assessing UE asymmetry caused by nerve injuries or central neurologic conditions. Hence, it is necessary to investigate the available literature and identify the present gaps in knowledge and redirect research focus onto such gaps.

The purpose of this review is to document the various tests/techniques/sensors used in clinical practice to assess UE asymmetry in a population with neurological conditions/injuries. This work was undertaken with the following goals:

1. Report trends in studies published from 2000 to 2022.
2. Classify the most widely used tests/techniques/sensors to assess UE asymmetry.
3. Classify the most widely studied neurological conditions/injuries.
4. Analyze the frequency of RWE based approaches compared to in-clinic approaches.

## Methods

The PRISMA-ScR guidelines for reporting scoping reviews were followed [41, 42]. The project was registered with the Open Science Framework (https://doi.org/10.17605/OSF.IO/8PFUW). A protocol was created and followed for the review (https://osf.io/bk3at).

### Literature search

Initial searches were performed on 1/26/2023 in PubMed, Scopus, and Web of Science. Date limits were set from 1/1/2000 forward. A follow-up search of multiple databases was performed on April 18, 2023. Results were also limited to 1/1/2000 forward. Databases for the follow up search were Ovid MEDLINE(R) (1946 + including epub ahead of print, in-process, and other nonindexed citations), Ovid Embase (1974 +), Ovid Cochrane Central Register of Controlled Trials (1991 +), Ovid Cochrane Database of Systematic Reviews (2005 +), and Scopus via Elsevier (1970 +). The initial search was performed by the study investigator SGB. The expanded and updated search strategies were designed and conducted by a medical librarian with input from the study investigators (SGB, AYS, KRK). The searched articles were limited to a publishing date between 1/1/2000 to 12/31/2022. Controlled vocabulary supplemented with keywords was used for this search. The actual strategies for each search that lists all search terms and how they are combined is available in the Table 1. Results included journal articles and peer reviewed conference proceedings in the English language and articles with available English translation. Duplicates were identified and removed from the main list using Endnote X9 (Clarivate, Philadelphia, PA).

**Table 1:** Search strategies

Initial search performed on 1/26/2023 by study investigators	
*PUBMED*	
(((((upper extremity[Title/Abstract]) OR (upper limbs[Title/Abstract])) OR (arm[Title/Abstract])) OR (hand[Title/Abstract])) AND (((activity[Title/Abstract]) OR (movement[Title/Abstract])) OR (motion[Title/Abstract]))) AND (((asymmetry[Title/Abstract]) OR (imbalance[Title/Abstract])) OR (inequality[Title/Abstract]))	
*WEB OF SCIENCE*	
(((((TS = (upper extremity) OR TS = (upper limbs)) OR TS = (arm)) OR TS = (hand)) AND ((TS = (activity) OR TS = (movement)) OR TS = (motion))) AND ((TS = (asymmetry) OR TS = (imbalance)) OR TS = (inequality)))	
*SCOPUS*	
TITLE-ABS-KEY ( "upper extremity" OR "upper limbs" OR arm OR hand) AND TITLE-ABS-KEY ( activity OR movement OR motion) AND TITLE-ABS-KEY ( asymmetry OR imbalance OR inequality)	
*Follow-up searches performed April 18, 2023 by medical librarian*	
*OVID* Database(s*): Ovid MEDLINE(R) 1946 to Present and Epub Ahead of Print, In-Process & Other Non-Indexed Citations and Ovid MEDLINE(R) Daily, EBM Reviews—Cochrane Central Register of Controlled Trials* March 2023, *EBM Reviews—Cochrane Database of Systematic Reviews* 2005 to April 18, 2023, *Embase* 1974 to 2023 April 17	
**#**	*Searches*
1	Functional Laterality/ or (asymmet* or symmetr*).ti,ab,kf. or (unilateral* or unimanual or bimanual or "right-dominan*" or "left-dominan*" or sided or side or "dominant-side" or "non-dominant" or nondominant or "left–right" or affected or non-affected or unaffected or hemi* or isometric* or ipselateral or ipsilesional or imbalanc* or laterality).ti
2	exp Upper Extremity/ or (arm or arms or forearm or elbow* or shoulder* or wrist or trunk or torso or hand or hands or grip or grasp* or "upper-limb*" or "inter-limb" or "upper-extremit*" or finger or fingers).ti
3	exp Motor Activity/ or Motor Skills/ or Movement/ or exp Muscle Strength/ or exp Neurologic Manifestations/ or (motion or movement* or moving or motor or kinematic* or "manual-skill" or "manual-dexterity" or dexterity or "sensory-motor" or "motor-deficit*" or dexterity or kinematic or activity or function or actigraph* or coordinat* or "muscle strength" or performance or impairment or impaired or inhibition or jerk or useage or function* or disuse or task or tasks).ti
4	"Brachial Plexus Neuropathies"/ or Brachial Plexus/ or Cerebral Palsy/ or Hemiplegia/ or "Gait Disorders, Neurologic"/ or exp Parkinsonian Disorders/ or ("pan-plexus" or "cerebral palsy" or spastic or hemiparesis or "multiple-sclerosis" or MS or parkinson* or "brachial-plexus" or "spinal-cord injur*" or stroke or poststroke or neurologic* or (nerv* adj2 (injur* or damag*))).ti
5	Psychomotor Performance/ or Actigraphy/ or Wearable Electronic Devices/ or "Monitoring, Physiologic"/ or Disability Evaluation/ or exp Artificial Intelligence/ or (actigraph* or measur* or test* or assess* or wearable* or sensor or sensors or scor* or index* or indeces or tool or tools or performance or evaluat* or detect* or classif* or quantif* or model* or analy* or "machine learning" or algorithm* or "artificial-intelligence").ti,ab,kf
6	and/1–5
7	(conference abstract or conference review or editorial or erratum or note or addresses or autobiography or bibliography or biography or blogs or comment or dictionary or directory or interactive tutorial or interview or lectures or legal cases or legislation or news or newspaper article or patient education handout or periodical index or portraits or published erratum or video-audio media or webcasts).mp. or conference abstract.st
8	6 not 7
9	limit 8 to english language [Limit not valid in CDSR; records were retained]
10	limit 9 to yr = "2000 -Current"
11	remove duplicates from 10
*SCOPUS*	
**#**	*Searches*
1	TITLE-ABS-KEY (asymmet* OR symmetr*) OR TITLE (unilateral* OR unimanual OR bimanual OR "right-dominan*" OR "left-dominan*" OR sided OR side OR "dominant-side" OR "non-dominant" OR nondominant OR "left–right" OR affected OR non-affected OR unaffected OR hemi* OR isometric* OR ipselateral OR ipsilesional OR imbalanc* OR laterality)
2	TITLE ( arm OR arms OR forearm OR elbow* OR shoulder* OR wrist OR trunk OR torso OR hand OR hands OR grip OR grasp* OR "upper-limb*" OR "inter-limb" OR "upper-extremit*" OR finger OR fingers)
3	TITLE ( motion OR movement* OR moving OR motor OR kinematic* OR "manual-skill" OR "manual-dexterity" OR dexterity OR "sensory-motor" OR "motor-deficit*" OR dexterity OR kinematic OR activity OR function OR actigraph* OR coordinat* OR "muscle strength" OR performance OR impairment OR impaired OR inhibition OR jerk OR useage OR function* OR disuse OR task OR tasks)
4	TITLE-ABS-KEY ( "pan-plexus" OR "cerebral palsy" OR spastic OR hemiparesis OR "multiple-sclerosis" OR ms OR parkinson* OR "brachial-plexus" OR "spinal-cord injur*" OR stroke OR poststroke OR neurologic*) OR TITLE-ABS-KEY ( nerv* W/2 ( injur* OR damag*))
5	TITLE-ABS-KEY ( actigraph* OR measur* OR test* OR assess* OR wearable* OR sensor OR sensors OR scor* OR index* OR indeces OR tool OR tools OR performance OR evaluat* OR detect* OR classif* OR quantif* OR model* OR analy* OR "machine learning" OR algorithm* OR "artificial-intelligence")
6	1 AND 2 AND 3 AND 4 AND 5
7	INDEX(embase) OR INDEX(medline) OR PMID(0* OR 1* OR 2* OR 3* OR 4* OR 5* OR 6* OR 7* OR 8* OR 9*)
8	( LIMIT-TO ( DOCTYPE, "ar")) AND ( LIMIT-TO ( LANGUAGE, "English")) AND ( LIMIT-TO ( SRCTYPE, "j"))

### Eligibility criteria

A thorough review of the literature was performed based on the following criteria.

Inclusion criteria:

- The study involved analysis of the dissimilarities in activity between the upper extremities (right vs. left) and included any assessment method to analyze this dissimilarity.
- The study involved subjects with neurological conditions/injuries.

Exclusion criteria:

- The article was a systematic review, a case study, or a book chapter.
- The study included only unimpaired subjects (No treatment group).
- The study involved analysis of the UE during indirect periodic tasks (e.g., gait, etc.).
- The article did not focus on the asymmetry between the upper extremities (e.g., device design and validation articles).
- The study was performed on newborns or infants.
- The article explored the UE activity via mathematical modelling.

### Selection process

**Fig. 1:**
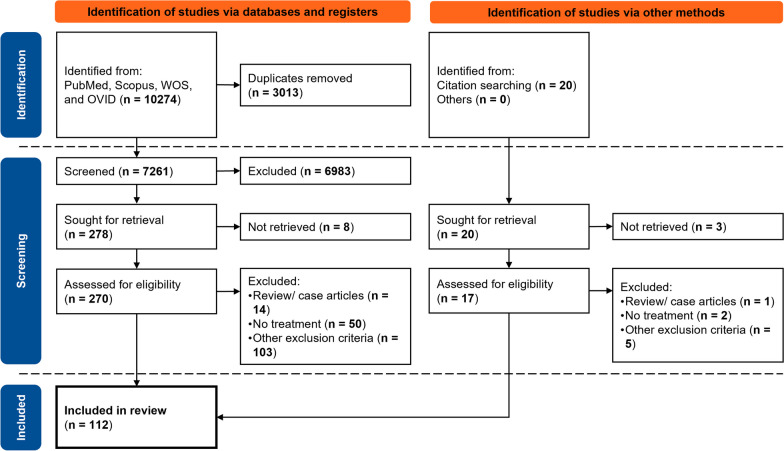
PRISMA flowchart for the literature search and exclusions. “n” is the number of articles in each given step

The selection process included several steps (Fig. 1). Database search results (publication years 2000 forward) were checked for duplicates. These unique articles were then screened using their abstracts for relevance to the review topic. Full texts for the screened articles were accessed online. The articles that could not be retrieved (for any reason) were disregarded from the review. The retrieved articles were assessed and selected based on the eligibility criteria. Several systematic review articles [36–39] related to the current review topic were identified during the search. The references listed in these review articles were screened and the process described above was performed on these references to check for eligibility.

### Data extraction

Selected articles were reviewed and the following data were extracted as per the protocol: study population, target condition/injury, types of tests performed, sensors used, real-world data collection as described in [35], outcome measures, and study results. Statistical significance was recorded. Data collected was classified as either objective, subjective, or a mixture of both. The search and mark functionality were used on pdf reader applications such as Adobe acrobat reader DC v2022.001.20142 (Adobe, San Jose, CA, USA) and Notability v11.3.1 (Ginger Labs, San Francisco, CA, USA). The extracted data were charted in an excel sheet (Excel, Microsoft 365 apps for enterprise, version 2211, Microsoft corporations, Redmond, WA, USA), and the counts were plotted using R version 4.2.0.

### Critical appraisal

A modified Sackett’s Level of Evidence (LoE) [43] was used to judge the quality of the studies based on the information provided in the articles. Level I indicated the study was a randomized controlled trial (RCT), Level II were cohort studies, Level III were case-controlled studies, and Level IV were poorly designed case-controlled studies. The LoE value indicating quality of the articles decreased from Level I to IV.

All the above steps were performed by SGB under the supervision of AYS and KRK.

## Results

### Selection of sources

From a total of 7281 unique articles, 112 met the specified criteria and were included in this review. Details of the exclusions performed are listed in Fig. 1. Review articles or case studies [36–39, 44–53] and studies without a treatment population (conducted exclusively on healthy subjects) [54–105] were excluded. Eleven articles studied UE asymmetry during indirect periodic movements (e.g., gait, etc.) [106–121]. Some excluded studies were designed to assess a specific device’s design [122–125], focused on mathematical modelling and analysis of the asymmetries [111, 126–130], or did not study the UE asymmetry resulting from any specific neurological condition (e.g., [131–151]). Articles focused on the reliability or validity of methods were also excluded [152–163]. Exclusions were also performed due to the subject population studied (Amputees [164, 165], and newborns/toddlers [166–171]). The study population, objectives and significant results from the reviewed articles are listed in Table 2.

**Table 2:** Summary of the articles selected for review (sorted by the target condition and first author’s last name)

Author	Study population (n; mean ± S.D. age)	Objective	Results (related to UE asymmetry)	Sackett’s level of evidence
*Brachial plexus injury*				
Duff et. al.; 2007 [172]	Patients with a BPI (16; 7.8 years); No healthy control	Examine the glenohumeral and scapulothoracic joint contributions to arm elevation to better understand the interlimb differences in children with unilateral BPI	The involved limb displayed lower glenohumeral joint excursion than the non-involved limb. The scapulothoracic joint made a greater contribution to arm elevation than the glenohumeral joint only in the involved limb	Level IV
Nazarahari et. al.; 2021 [173]	Patients with a BPI (15; 54 ± 16 years); Healthy control (15; 24 ± 3 years)	Assess the alterations of shoulder motion pattern and trick trunk movements caused by BPI in terms of their impact on shoulder function asymmetry	AI from several kinematic scores of the upper arm and trunk showed a significant difference (P < .05) between the affected and control groups	Level III
Webber et. al.; 2019 [174]	Patients with a BPI pre-surgery (10; 34 ± 12 years), post-surgery (10; 35 ± 12 years); Healthy control (10; 38 ± 12 years)	Develop a method to report bilateral UE activity in patients with BPI who have undergone surgery to restore arm function	Compared to controls, the pre-surgery group (p < 0.0001, p < 0.0001) and post-surgery group with pan-plexus injuries (p = 0.0074, p = 0.0242) both exhibited statistically significant differences in forearm and upper arm asymmetry, respectively	Level II
*Cerebral palsy*				
Beani et. al.; 2019 [175]	Unilateral CP (50; 9.93 ± 5.23 years); Healthy control (50; 10.14 ± 5.19 years) children	Assess the feasibility of comprehensive indices derived using 3D accelerometers to gauge the use of UE between treatment and control groups while drawing comparisons with currently used outcome measures	AI (p < 0.001) was related to MACS levels and was different between the treatment and control groups (p < 0.001). Quantitative data better described UE activity and asymmetry	Level III
Dellatolas et. al.; 2005 [176]	Children with CP (30; 7.52 ± 0.33 years); Two groups of healthy control (30; 7.47 ± 0.26 years & 30; 7.52 ± 0.16 years)	Verify whether PMTs are useful for assessment of hand skills; Relate PMT to ADL	PMT times correlated well with ADL. Unimanual ADL are associated with the less affected hand and bimanual with the more affected hand	Level III
Friel et. al.; 2014 [177]	Children with unilateral CP (35; 7.9 ± 2.9 years); No healthy control	Examine the association between an estimate of CST dysgenesis, hand function, and bimanual training in children with CP	Bimanual training improved hand function (p < 0.001). Estimated CST dysgenesis correlated with hand function but not with its post-training improvement	Level II
Gaillard et. al.; 2018 [178]	Children with CP (23; 11.83 ± 2.67 years); Healthy control (23; 11.92 ± 2.42 years)	Evaluate the relationship between the movement abnormalities of the impaired upper limb in children with unilateral CP and bimanual performance	Values of kinematic indices were significantly higher in children with unilateral CP than in typically developing children	Level III
Gordon et. al.; 2007 [179]	Children with hemiplegic CP (10; 8.43 ± 3.67 years); Healthy control (10; 6.76 ± 2.33 years)	Examine the efficacy of HABIT in improving bimanual coordination in children with CP	Children in the intervention group showed improved bimanual coordination (p < 0.05)	Level I
Hoyt et. al.; 2020 [180]	Children with CP (26; 8.5 years); Healthy control (26; 8.4 years)	Use accelerometry to measure motor behavior. Compare accelerometry to clinical assessment	Hemiparesis is associated with a lower use ratio than controls (p < 0.001). Children with hemiparesis depended largely on their dominant UE for independent movements	Level III
Huang et. al.; 2014 [181]	Children with CP (9; 4 years); Healthy control (9; 3.92 years)	Examine body-scaled information that specifies the reach patterns of children with hemiplegic cerebral palsy and children with typical development	The critical ratio was not significantly different for either preferred or nonpreferred arms within and between groups. All children used an exclusive 2-handed reach at a similar dimensionless ratio	Level IV
Hung et. al.; 2004 [18]	Teenagers with CP (10; 13.42 ± 3.5 years); Healthy control (10; 13.08 ± 2.92 years)	Examine bimanual coordination using a drawer-opening task under speed and hand constraints	The children with hemiplegic CP were slower (p < 0.001) and less coordinated in this task, with reduced movement overlap of the two hands (p < 0.001) and sequential completion of the two movement objectives (p < 0.001). The hand used for each task affected task performance (p < 0.05)	Level IV
Hung et. al.; 2020 [182]	Children with Unilateral CP (10; 9.5 ± 1.75 years) with healthy children (10; 9.67 ± 2.25 years); Healthy adults (10; 26.5 ± 7.08 years) as control	Examined how children with USCP perform a functional symmetric bimanual tray lifting task	Children with USCP exhibited greater bilateral asymmetry in hand vertical position, timing, upper arm, and elbow control than other groups (p < 0.05)	Level II
Kara et. al.; 2020 [183]	Children with CP (15; 12.4 ± 2.8 years); Healthy control (16; 12.3 ± 2.07 years)	Investigate performance (touch-coordinate errors, inter-touch interval) of touch screen technology in adolescents with unilateral CP and healthy peers	When comparing the dominant and non-dominant hand in the CP group, there was a significant difference on touch coordinate error with no visual feedback (p = 0.01)	Level III
Klevberg et. al.; 2018 [184]	Children with CP (102; 2.38 years); No healthy control	Investigate development of bimanual performance among young children with unilateral or bilateral CP	Children with symmetric hand use performed better than those with asymmetric hand use (p = 0.003)	Level II
Langan et. al.; 2010 [185]	Patients with CP (11; 33 ± 10 years); Healthy control (10; 32 ± 9 years)	Examine movement time and kinematic properties of unilateral and bilateral reaching movements in adults with CP	Bilateral simultaneous reaching movements were temporally and spatially coupled. Movement of the less affected arm slowed to match the movement of the more affected arm	Level III
McCall et. al.; 2022 [186]	Children with hemiplegic CP (4; 11.25 ± 1.5 years); Healthy control (10; 11.8 ± 1.6 years)	Assess and compare finger force and movement individuation in children with hemiplegic CP	Individuated force and movement were substantially reduced in the paretic versus non paretic hands of children with hemiplegic CP (p < 0.001)	Level IV
Mutalib et. al.; 2019 [187]	Children with CP (15; 8.7 ± 2.7 years); Healthy children as control (17; 8.2 ± 2.5 years)	Examine temporal, force, and kinematic coordination between the two hands in USCP affected and typically developing children, during a physically coupled lifting task	USCP subjects displayed compensating strategies for inter-limb asymmetries, such as muscle strength	Level III
Ricken et. al.; 2005 [188]	Children with spastic hemiparetic CP (10; 8.6 ± 1.8 years); No healthy control	Examine coordination of reaching with the impaired and non-impaired arm in children with spastic hemiparetic CP	Coordination patterns between elbow, shoulder, and trunk displayed less similarity when reaching with the impaired arm compared to the non-impaired arm	Level IV
Shum et. al.; 2020 [189]	Children with CP (5; 17 ± 3 years); Healthy children (12; 17 ± 3 years)	Explore the viability of an immersive VR environment that manipulated visual feedback (i.e., EA) during bimanual movements that could be implemented in engaging home-based rehabilitative systems	EA improved symmetry in the control and treatment groups	Level IV
Smits-Engelsman et. al.; 2005 [190]	Children with CP (20; 9.58 ± 3.33 years); 20 age-matched healthy control (no demographics data)	Measure the capacity of one group of spastic muscles (the finger flexors) to exert force and to grade force	Results showed that force generated with the affected hand was only one-third of that generated by the non-affected hand (p < 0.001) with slower time to peak (p < 0.001)	Level III
Steenbergen et. al.; 2000 [191]	Patients with Spastic Hemiparesis (CP) (6; 17.3 ± 1.6 years); No healthy control	Examine the limb dynamics of both limbs of spastic hemiparetic subjects under various task constraints	Peak velocity was not different with respect to the hand used. The time to reach peak velocity was different under unimanual and bimanual responding (p < 0.01). The analysis of mean velocity showed a higher mean velocity of the unimpaired hand as compared to the impaired hand (p < 0.05)	Level IV
Steenbergen et. al.; 2004 [192]	Teenagers with CP (6; 17.25 ± 1.25 years); No healthy control	Investigate ipsi- and contra-lesional control of prehension after unilateral brain damage	Kinematic variables of the transport and grasp component were remarkably similar between both sides of the body	Level IV
Steenbergen et. al.; 2008 [193]	Children with hemiplegic CP (7; 8.97 ± 3.14 years); No healthy control	Examine unimanual and bimanual fingertip force control during grasping in children with hemiplegic CP	The less-affected hand slowed down when moving together with the affected hand. Grip force at onset of load force, and peak grip force improved marginally for the affected hand during bimanual tasks	Level IV
Tomhave et. al.; 2014 [194]	Children with CP (37; 9.8 years); No healthy control	Determine how the affected hemiplegic hand and contralateral dominant hand in children with hemiplegic cerebral palsy compare with age-matched norms for grip strength, pinch strength, and dexterity	Affected hands had significantly less grip and pinch strength than the contralateral hands (p < 0.001). Dexterity in both affected and contralateral hands was significantly less than normative values (p < 0.01)	Level II
van Roon et. al.; 2000 [195]	Teenagers with CP (11; 17.1 ± 1.2 years); No healthy control	Locate the source of slowness in typing movements in subjects with spastic hemiparesis and to examine whether enlargement of keys would facilitate typing	The impaired hand tapped slower and more irregularly and exerted less force (p < 0.05). Enlargement of the keys shortened flight time (i.e. time in which the finger moves through the air from one key to the next) of the impaired hand, but not dwell time (p < 0.01)	Level IV
van Thiel et. al.; 2000 [196]	Teenagers with hemiparetic CP (12; 17.37 ± 1.3 years); Healthy control (12; 23 years)	Examine the control that hemiparetic subjects have over fast, unimanual aiming movements	Mean movement velocity was lower for the CP subjects than for the control subjects (p < 0.05). Path variability was larger for the CP group (p < 0.05)	Level III
van Thiel et. al.; 2001 [197]	Children with CP (8; 18 ± 1.5 years); No healthy control	Examine the degree and timing of shoulder displacements during hitting, reaching, and grasping movements performed by young adults with hemiparetic cerebral palsy	The hand used was significant for reaching and grasping tasks (p < 0.05). the impaired hand was less fluent compared to the unimpaired hand (p < 0.05)	Level IV
Verrel et. al.; 2008 [198]	Patients with CP (6; 20 to 25 years); Healthy control (10; 14 to 19 years)	Investigated eye–hand coordination in adolescents with hemiparetic CP and neurologically healthy controls	Increase in visual monitoring of participants with CP when using their affected hand at the beginning as well as during object transport	Level IV
Volman et. al.; 2005 [199]	Children with hemiplegic CP (10; 10.6 ± 1.2 years); No healthy control	Investigate the effect of amplitude incongruence and form incongruence on the performance of the affected and non-affected arm	Aspects of movement execution, but not aspects of movement planning are affected by the “hemiplegic” condition in children with hemiplegic CP	Level IV
Xu et. al.; 2015 [200]	Children with CP (68; 4.58 ± 2.75 years); No healthy control	Investigate the characteristics of motor unit recruitment and coordination function of the wrist during maximum isometric voluntary contraction in children with hemiplegic CP	The activation in the affected hand were higher than that of the unaffected hand (P < .001). The co-contraction ratio of the unaffected hand was significantly lower than that of the affected hand (P < .001)	Level II
*Multiple sclerosis*				
Pau et. al.; 2021 [201]	Patients with MS (28; 50.6 ± 9.3 years); Healthy control (28; 48.7 ± 9.5 years)	Characterize the major features of upper limb usage in patients with MS with respect to unaffected individuals, in terms of overall activity time, intensity, and inter-limb symmetry in uni- and bilateral activities	The MS group were characterized by significantly lower overall activity, they used their dominant limb for a significantly longer time and, while performing bilateral activities, their dominant limb expressed movements of superior intensity in a proportion higher than what was observed in unaffected individuals	Level III
Severijns et. al.; 2016 [202]	Patients with MS (19; 56 ± 12 years); Healthy control (19; 56 ± 13 years)	Determine the difference in hand grip fatigability between healthy persons and people with MS for both hands during low-intensity hand grip exercises	There was a significant decrease in maximal hand grip strength after exercising in both groups and for both hands (p < 0.01)	Level III
Solaro et. al.; 2020 [203]	Patients with MS (549; 45 ± 13.14 years); No healthy control	Determine whether a non-linear association between NHPT performance and EDSS, conditional on the level of asymmetry between the two hands exists	It was demonstrated that by including information about asymmetry in the model, an average positive change in accuracy of 0.16 EDSS points (SD = 0.49) was obtained in the overall sample (n = 549); additionally, an average change in accuracy was highest among patients with EDSS > 6, followed by patients with EDSS ≤ 3, while average change is negligible in patients with EDSS in the 3.5 – 6 range	Level IV
Unluer et. al.; 2019 [204]	Patients with MS (20; 39.95 ± 12.22 years); Healthy control (20; 35.99 ± 11.96 years)	Investigate upper extremity function and shoulder position sense in patients with MS	Upper extremity function scores were lower and shoulder position sense error scores were greater in patients with multiple sclerosis in comparison to healthy controls (p < .05)	Level III
*Parkinson’s disease*				
Di Caprio et al.; 2020 [205]	Patients with right-dominant PD (17; 57.6 ± 6.8 years), left-dominant PD (17; 61.6 ± 5.6 years); Healthy control (24; 58.5 ± 6.9 years)	Assess whether PD patients show global or selective impairment in inhibition and compare right dominant patients with left dominant patients	Reactive inhibition was more impaired in PD patients (p < 0.05). Proactive inhibition was not different. No difference between right and left dominant PD patients	Level III
Amano et. al.; 2015 [206]	Patients with PD (14; 64 ± 13 years); Healthy control (4; 56 ± 11 years)	Assess the effects of dedifferentiation in the PD population using a reaching task	The dominant arm became slow and less flexible making its movement dynamics similar (p < 0.05) to the non-dominant arm	Level IV
Gorniak et. al.; 2013 [207]	Patients with PD and DBS implant (10; 61 ± 8 years); No healthy control	Investigate the effects of subthalamic DBS on time and force characteristics of simulated ADL	The data indicated that DBS parameter state did not affect most aspects of fine motor control in ADL-like tasks; however, features such as increased grip force and grip symmetry varied with the stimulation state. In the absence of DBS parameters, patients exhibited significant grip force asymmetry	Level II
Ham et. al.; 2015 [208]	Patients with PD affecting their dominant-side (57) and non-dominant-side (61) (64.7 ± 7.7 years); No healthy control	Compare dominant-side onset and non-dominant-side onset PD to evaluate whether dominant-side onset PD has greater neural reserve and fewer motor deficits despite similar pathological changes	Dominant-side subjects had fewer motor deficits than non-dominant-side subjects (p < 0.01). Hence, non-dominant-side PD causes higher UE asymmetry	Level III
Jackson et. al.; 2000 [209]	Patients with PD (14; 64.17 ± 5.76 years); Healthy control (4; 66 years)	Examine the proposal that the basal ganglia may serve to scale the amplitude of limb movements, with basal-ganglia dysfunction leading to the inappropriate scaling of intended motor activity (opening of hand)	Patients opened their hands wider for unimanual tasks compared to bimanual tasks (p < 0.05) with their non-preferred hand opening les wide (p < 0.05)	Level IV
Louie et. al.; 2009 [210]	Patients with PD (85; 60.9 ± 11.1 years); Healthy control (21; 57.9 ± 8.3 years)	Examine whether quantitative measures of movement velocity showed a relationship between bradykinesia and disease severity and whether this relationship was the same on the more affected and less affected sides	The velocities of finger and arm movements on both sides were inversely correlated with disease severity (p < 0.03). The performance of the less affected side was not different from that of controls (p > 0.05), whereas that of the more affected side was slower (p = 0.019)	Level II
Schaefer et. al.; 2021 [211]	Patients with PD (22; 63.58 ± 10.26 years); Healthy control (25; 68.48 ± 6.81 years)	Investigate whether mechanical muscular oscillations differ between PD patients and controls	Significant differences appeared for the power–frequency-ratio (p = 0.001). PD patients showed altered mechanical muscular output compared to controls	Level III
Stegemoller et. al.; 2016 [212]	Patients with PD (41; 68 ± 11 years); No healthy control	Compare repetitive finger movement performance between the more and less affected side, and the difference in clinical ratings among performance groups	There were no significant differences between the more and less affected sides	Level II
Yang et. al.; 2020 [213]	Patients with PD (16; 62.8 ± 6.83 years); Healthy control (25; 59.13 ± 6.38 years)	Investigate EMG characteristics of the UE between PD patients and healthy control subjects	There was a significant difference in the duration of activation and recurrence rate between the sides in the PD group (p < 0.05)	Level III
*Peripheral nerve injury*				
Philip et. al.; 2021 [214]	Patients with a PNI affecting the dominant side (22; 42.23 ± 15.53 years) & non-dominant side (26; 46.27 ± 15.63 years); No healthy control	Identify how individuals respond to unilateral UE PNI via compensation (increased use of the nondominant hand)	Hand usage (dominant/nondominant) in the treatment group did not differ from typical adults, regardless of injured side (p > .07). Compensation was associated only with dominant hand dexterity (p < 0.001), not on nondominant hand dexterity, rehabilitation, or other patient and/or injury factors (p > 0.1)	Level II
*Spinal cord injury*				
Bondi et. al.; 2022 [215]	Patients with a SCI (73; 49.9 ± 19.9 years); No healthy control	Determine whether cerebral dominance influences upper extremity recovery following cervical SCI	There were no significant differences (p < 0.05) for observed and relative recovery, between the dominant and non-dominant upper extremities	Level II
Britten et. al.; 2017 [216]	Patients with a SCI (20; 48.1 ± 15.9 years); Young healthy control (16; 23.68 ± 4.54 years) and old healthy control (16; 70.92 ± 7.2 years)	Investigate unimanual and bimanual coordination in patients with acute SCI using 3D kinematic analysis as they performed naturalistic reach to grasp actions with one hand, or with both hands together	Participants with a SCI produced reach-to-grasp actions which took longer, were slower, and had longer deceleration phases than uninjured participants	Level II
Calabro et. al.; 2016 [217]	Patients with SCI (16; 54.3 ± 12.8 years); Healthy control (20; 48.4 ± 18.1 years)	Study the effect of abnormalities in movement kinematics and EMG during bilateral reach-to-grasp movements	The more impaired arm affects the less impaired arm, negatively, in humans with asymmetric functional impairments in the arms due to incomplete cervical SCI	Level III
Lei et. al.; 2018 [218]	Patients with a SCI (20; 48.1 ± 15.9 years); Healthy control (20; 42.7 ± 10.7 years)	Study the different phases of unilateral self-paced reach-to-grasp movements in the more and less affected arms of individuals with cervical SCI and in age-matched controls	Deficits in movement kinematics during reach-to-grasp movements are more pronounced at the time to close the hand in the more affected arm of SCI participants	Level III
*Schizophrenia*				
Lisi et. al.; 2018 [219]	Patients with SCZ (14; 30.78 ± 6.26 years); Healthy control (14; 30.9 ± 15.5 years)	Characterize asymmetries in movement initiation in SCZ by exploring single actions	SCZ patients, besides being overall slower than controls, additionally presented with a bias affecting both the moving hand and the side from which movements were initiated	Level III
*Stroke*				
Akremi et. al.; 2021 [220]	Patients affected by stroke (19; 61.6 ± 14.4 years); Healthy control (20; 51 ± 14.9 years)	Assess kinematic and kinetic parameters for upper limb co-ordination during a bilateral pushing task	The paretic side displayed considerable lag (p < 0.01) and lower forces (p < 0.01) than the non-paretic side	Level IV
Assadi et. al.; 2022 [221]	Patients affected by stroke (82; 71 years) assessed three times post stroke; No healthy control	Assess longitudinal changes in function, dexterity, grip strength, and self-perception of the less-affected UE and determine the association of both UEs to ADL during the first 6 months poststroke	Dexterity improved significantly in 6 weeks (P < 0.001) and grip strength improved significantly between 6 weeks and 6 months post-stroke (p < 0.01)	Level II
Bailey et. al.; 2015 [222]	Patients affected by stroke (48; 59.7 ± 10.9 years); Healthy control (74; 54.3 ± 11.3 years)	Quantify real-world bilateral upper-limb activity in nondisabled adults and adults with stroke using a recently developed accelerometry-based methodology	Nondisabled adults demonstrated equivalent use of dominant and nondominant upper limbs. Bilateral UE activity intensity was lower (P < .001) and more lateralized in adults with stroke (P < .001)	Level I
Bailey et. al.; 2015 [223]	Patients affected by stroke (46; 60 ± 11 years); No healthy control	Characterize affected UE activity and examine potential modifying factors of affected UE activity in community-dwelling adults with chronic stroke	Hours of affected and unaffected UE activity were strongly correlated. Increased severity of motor dysfunction and dependence in ADLs were associated with decreased affected UE activity	Level I
Basilio et. al.; 2021 [224]	Patients affected by stroke (14; 54 ± 11 years); Healthy control (12; 55 ± 9 years)	Investigate UE energy demand during unilateral arm crank submaximal exercise testing in individuals with stroke compared with healthy controls	The energy demand of the paretic side compared with the nonparetic side of the stroke group was greater than the dominant compared with the nondominant side of the control group (p = 0.005)	Level III
Bertrand et. al.; 2003 [225]	Patients affected by stroke (15; 52 ± 12.4 years); Healthy control (15; 47.7 ± 11.5 years)	Verify whether subjects with hemiparesis produced asymmetrical forces during a bilateral submaximal grip task and whether this asymmetry is related to weakness of the paretic limb	In subjects with hemiparesis, the force ratios in the bilateral task were related to the ratios of maximal voluntary forces (p < 0.013). Severely weak hemiparetic subjects produced lower force ratios than mildly weak hemiparetic subjects and healthy subjects (p < 0.001)	Level III
Biryukova et. al.; 2022 [226]	Patients affected by stroke (24; 55.41 ± 10.84 years); No healthy control	Study the dependence of poststroke motor impairments of paretic and non-paretic arm on lesion lateralization, and paresis severity	The joint torques of the non-paretic arm were greater in the case of left hemispheric lesions, characterized by more pronounced asymmetry of joint torques than in right hemispheric lesions	Level IV
Buxbaum et. al.; 2001 [227]	Patients with a Unilateral Right Hemisphere Stroke (8; 66.8 ± 11.9 years); Healthy control (12; 60.2 ± 12.6 years)	Study that, for a reaching task, a left–right asymmetry in treatment group is dependent on distractor stimuli, which competes with the target for control of action, Hence, increasing motor response time in the patients	Treatment group showed slower response due to distractors on the right of the hand, but faster response due to distractors on the left (p < 0.05). Left–right selection asymmetry in neglect may be hand centered	Level III
Cai et. al.; 2019 [228]	Patients affected by stroke (15; 57 ± 11 years); Healthy control (15; 57 ± 10 years)	Determine whether stroke affected how accurately individuals with stroke perceive their self-generated torques during a single-arm task	Participants matched torques at each elbow, for each target torque and movement direction, with a similar accuracy and precision to controls, regardless of the arm tested (p > 0.050)	Level III
Calautti et. al.; 2006 [229]	Patients affected by stroke (20; 61 ± 10 years); Healthy control (20; 58 ± 10 years)	Quantify the ability of stroke patients to finger tap in rhythm with auditory cues given at physiological rate	Regularity Index of their affected hand was significantly worse compared to their unaffected hand and to age-matched controls (p < 0.05 and p < 0.01, respectively)	Level III
Chae et al.; 2002 [230]	Patients affected by stroke (26; 51 ± 15.6 years); No healthy control	Describe the relationship between poststroke UE muscle weakness and cocontraction, and clinical measures of upper limb motor impairment and physical disability	The strength of muscle contraction was significantly stronger in the nonparetic limb (p < 0.001). The strength of muscle contraction in the paretic limb correlated significantly with the clinical measures	Level IV
Dash et. al.; 2019 [231]	Patients affected by stroke (8; 43 ± 8.15 years); Healthy control (12; 35 ± 8 years)	Investigate the measure of post-stroke spasticity affecting grip-strength through quantification of interaction between antagonist and agonist muscles using complexity analysis of EMG signals during isometric grip in healthy and post-stroke participants	EMG signals were significantly different (p < 0.05) between paretic and non-paretic hands of post-stroke participants	Level III
de Lucena et. al.; 2017 [232]	Patients affected by stroke (9; 56.1 ± 14.9 years); No healthy control	Characterize the relationship between kinematic and conventional measures of jerk and acceleration asymmetry, and UE recovery in the treatment group	The non-affected limb showed a significantly greater activity time ratio (p < 0.001), lower average acceleration (p < 0.01), and lower average jerk magnitude (p < 0.01)	Level IV
de Niet et. al.; 2007 [233]	Stroke patients with FMA < 45 (8; 56.1 ± 13 years), FMA > 45 (10; 52.1 ± 13.9 years); Healthy control (5; 43 ± 13.2 years)	Evaluate the Stroke Upper-Limb Activity Monitor to measure the amount of upper limb usage to differentiate between the two treatment groups and the control groups	The level of usage of the affected upper limb of stroke patients was lower than that of the nondominant upper limb of control subjects (electrogoniometry p < 0.01; accelerometry p < 0.01). Stroke patients had higher asymmetry than control subjects in both electrogoniometry (p < 0.01) and accelerometry (p < 0.01). Well-recovered stroke patients had significantly lower asymmetry compared with moderately recovered patients on both electrogoniometry and accelerometry	Level III
Gebruers et. al.; 2008 [234]	Patients affected by stroke (39; 73.8 ± 10 years); No healthy control	Investigate disuse of the impaired arm in acute stroke patients using actigraphy	Actigraphy was able to reliably discriminate less impaired from more impaired stroke patients (p < 0.001). Actigraphy was moderately correlated with AHA (positive) and NIHSS (negative)	Level II
Gosser et. al.; 2015 [235]	Patients affected by stroke (12; 57.75 ± 8.48 years); Healthy control (13; 58.62 ± 8.39 years)	Investigate movement efficiency in people with and without stroke during both unimanual and bimanual upper extremity reaching tasks	The unimpaired limb accommodated its movements to that of the less efficient paretic limb during bimanual conditions. The impaired limb’s performance did not differ between the unimanual and bimanual conditions (P > 0.05)	Level III
Gurari et. al.; 2017 [236]	Patients affected by stroke (14; 57.79 ± 7.04 years); Healthy control (9; 56.2 ± 6.8 years)	Investigate whether individuals with chronic stroke who have impairments mirroring arm positions also have impairments identifying the location of each arm in space	Participants with stroke had greater errors than the controls in both their paretic and non-paretic arm when matching positions during passive movements; yet stroke participants performed comparable to the controls during active movements	Level IV
Harris et. al.; 2006 [237]	Patients affected by stroke (93; 68.7 ± 9.4 years); No healthy control	Determine if UE impairment and function in individuals with chronic stroke is dependent upon whether the dominant or nondominant hand is affected	Affected side (dominant or nondominant) was a significant factor in UE function (p = 0.01)	Level II
Hollis et. al.; 2021 [238]	Children with stroke (30; 11.17 ± 3.83 years); Healthy control (23; 11.08 ± 4.42 years)	Evaluate the efficacy of bilateral actigraphy to quantify UE movement in children with perinatal stroke and HCP	Stroke participants demonstrated higher asymmetry. The strongest correlations were observed between the Block Ratio and the AHA and MA (r = 0.93 and 0.87 respectively)	Level III
Hu et. al.; 2007 [239]	Patients affected by stroke (8; 36 to 59 years); Healthy control (5; 23 to 31 years)	Investigate the properties of MMG, or muscle sound, of the paretic muscle in the affected side of hemiplegic subjects after stroke during isometric voluntary contractions	Both the MMG and EMG RMS values in the healthy and unaffected groups were found to be significantly higher than the affected group (P < 0.05)	Level IV
Iacovelli et. al.; 2019 [240]	Patients affected by stroke (20; 69.2 ± 10.1 years); Healthy control (17; 70.5 ± 7.3 years)	Verify if the actigraphic AI, as calculated by Rabuffetti, can identify the paretic arm of acute stroke patients; verify if such AI can properly quantify the clinical severity of acute stroke patients in the very particular environment of a stroke unit	Both asymmetry indices used were smaller in the paretic than in the unaffected arm (p = 0.004). Asymmetry rate index was greater in stroke patients than in controls and positively correlated with NIHSS total scores	Level III
Johnson et. al.; 2021 [241]	Patients affected by stroke (20; 62.3 ± 7.6 years); Healthy control (10; 566.8 ± 9 years)	Compare fine and gross motor hand dexterity of the ipsilesional hand post-stroke with controls, normative values, and the contralesional hand	Individuals with stroke demonstrated poorer performance with the ipsilesional arm relative to both the control group and the normative values. Ipsilesional arm performance was significantly better than performance with the contralesional arm	Level III
Johnson et. al.; 2022 [242]	Patients with a Unilateral RHD (15; 60.67 ± 1.8 years); Unilateral LHD (15; 64.2 ± 1.83 years); Healthy control (10; 60.2 ± 2.18 years)	Assess the effects of stroke in the right vs. the left hemisphere on how bimanual tasks modify affected and non-affected arm performance and coordination	Individuals with stroke displayed poor grasp and pick-up coordination compared to heathy controls (p < 0.05). The LHD group showed more deficits in grasp coordination	Level III
Kang et. al.; 2014 [243]	Patients affected by stroke (9; 64.2 ± 18.8 years); Healthy control (9; 67.6 ± 13.8 years)	Investigate force variability generated by both the paretic and non-paretic hands during bimanual force control	Greater bimanual force variability in the stroke group than the control group (p < 0.001) and increased force variability by the paretic hands during bimanual force control in comparison to the non-paretic hands (p < 0.001)	Level IV
Kang et. al.; 2017 [244]	Patients affected by stroke (9; 64.2 ± 18.8 years); Healthy control (9; 73.1 ± 5 years)	Examine bilateral synergies using the uncontrolled manifold approach while individuals in a chronic stage after stroke executed bilateral isometric force control at three submaximal force levels	Decreased bilateral synergies in patients with stroke as compared to controls at 50% of MVC	Level IV
Kantak et. al.; 2016 [245]	Patients affected by stroke (11; 59.63 ± 11 years); Healthy control (10; 61.2 ± 15.79 years)	Investigated how patients with chronic stroke and age-matched controls coordinate their arms while performing symmetric and asymmetric movements to accomplish common task goals compared to independent task goals	Individuals with stroke were less coordinated in space and time during common-goal bimanual actions employing asymmetric arm movements. patients demonstrated lesser contribution of their paretic arm compared to their non-paretic arm during common-goal conditions	Level IV
Kantak et. al.; 2016 [246]	Patients affected by stroke (14; 53 ± 15.4 years); Healthy control (10; 59.75 ± 23.06 years)	Determine bimanual coordination deficits in patients with stroke using 3-dimensional kinematic analyses as they perform naturalistic tasks requiring collaborative interaction of the 2 arms	Patients demonstrated an impaired ability to cooperatively interact their 2 arms for an efficient pickup, leading to longer pickup times	Level IV
Kim et. al.; 2016 [247]	Patients affected by stroke (19; 66.6 ± 6.5 years); Healthy control (19; 66.4 ± 4.8 years)	Investigate the effect of stroke-related constraints on multi-finger force control abilities in a visuomotor task	The impaired hand in the stroke survivors showed deficits in motor performance attributed mainly to lower accuracy and reproducibility as compared to control hands (p < 0.05)	Level III
Koesler et. al.; 2009 [248]	Patients affected by stroke (12; 67 ± 7 years); No healthy control	Investigate the effect of electrical somatosensory stimulation on motor performance of the affected hand	Somatosensory stimulation of the median nerve of the affected hand enhanced the frequency of index finger and hand tapping movements and improved the kinematics of reach-to grasp movements performed with the affected hand, compared with baseline (p < 0.01)	Level IV
Lai et. al.; 2019 [249]	Patients affected by stroke (22; 53.7 ± 9.8 years); Healthy control (16; 23.4 ± 3.4 years)	Analyze the relationship of bimanual force coordination control deficits in both hands with motor and functional performances of the paretic upper extremity in stroke patients	The alternating time from the non-paretic to the paretic hand shorter for stroke patients (p < 0.001). The grip force generated for coordination in the healthy group was significantly greater than that of the stroke group (p < 0.05)	Level III
Lakhani et. al.; 2017 [250]	Less (13; 69.62 ± 5.17 years) and More (9; 59.44 ± 6.96 years) impaired patients affected by stroke; No healthy control	Understand whether MWF in sensorimotor regions of interest is a biomarker of long-term impairment, function, or arm use in a population of individuals living with chronic stroke	There were no relationships between MWF asymmetry ratio and upper-limb use	Level II
Lang et. al.; 2008 [251]	Patients affected by stroke (52; 64 ± 14 years); No healthy control	Estimate minimal clinically significant difference values of several upper-extremity measures early after stroke	ARAT, out of all the measures tested, had the highest MCID score	Level II
Lang et. al.; 2007 [252]	Patients affected by stroke (34; 63.9 ± 14.8 years); Healthy control (10; 59 ± 13 years)	Determine the amount of UE use in people with hemiparesis post stroke during their inpatient rehabilitation stay	Hemiparetic subjects used their affected and unaffected upper extremities substantially less than control subjects; affected UE use is minimal during the inpatient rehabilitation stay	Level II
Lee et. al.; 2015 [253]	Patients affected by stroke (13; 65.64 ± 7.35 years); Healthy control (13; 65.59 ± 9.4 years)	Compare force modulation below 1 Hz in chronic stroke and age-matched healthy individuals	Force modulation below 1 Hz differentiated the stroke individuals and healthy controls (p < 0.01), as well as the paretic and non-paretic hands (p < 0.05). Similarly, the paretic hand exhibited greater power at 0.2 Hz, and lesser power at 0.6 Hz than the nonparetic hand	Level IV
Lee et. al.; 2020 [254]	Patients affected by stroke (29; 58.87 ± 11.83 years); No healthy control	Compare the differences in the amount of activity of the nondominant and dominant affected hands among patients with poststroke right hemiparesis	The asymmetry and differential activity of both hands were worse in the patients with poststroke right hemiparesis, whose dominant hand was affected	Level II
Lewis et. al.; 2004 [255]	Patients affected by stroke (9; 56 ± 12 years); Healthy control (9; 54 ± 9 years)	Examine intralimb coordination in a frequency-scaled task completed in unimanual and bimanual conditions	Trajectory variability was higher in the stroke group (p < 0.001). The impaired hand showed higher variability than the nonimpaired hand	Level IV
Li et. al.; 2003 [256]	Patients affected by stroke (16; 26 to 87 years); Healthy control (16; 25 to 86 years)	Investigate changes in finger interaction after stroke with strongly unilateral motor effects. Effects of age on finger interaction were also analyzed	Peak forces produced by the fingers of the impaired hand were about 36% less than those produced by the unimpaired hand (p < 0.001). Two-hand tasks were accompanied by an additional drop in the force of individual fingers	Level III
Lodha et. al.; 2012 [257]	Patients affected by stroke (12; 64.88 ± 8.07 years); Healthy control (12; 66.67 ± 9.39 years)	Investigate the influence of task constraints on bimanual force control strategies poststroke	The paretic hand contributed less force than the non-paretic hand in finger extension whereas this relationship was reversed in power grip. Reduction in motor impairments was associated with increased symmetry and coordination in bimanual tasks	Level III
Lodha et. al.; 2012 [258]	Patients affected by stroke (10; 64.87 ± 8.07 years); Healthy control (10; 66.66 ± 9.36 years)	Determine the asymmetry and coordination of force output during a bimanual isometric task with a common goal in chronic stroke relative to age-matched controls	The stroke group demonstrated greater asymmetry and reduced coordination in force produced by each hand. Bimanual force coordination increased at higher forces in controls but not in stroke	Level III
Maenza et. al.; 2020 [259]	Patients affected by stroke on the left (48; 60.28 years) and on the right (64; 59.12 years); Healthy control (54; 65.59 ± 9.4 years)	Examine whether functional motor deficits in the less-affected arm, measured by standardized clinical measures of motor function, also depend on the hemisphere that was damaged and on the severity of contralesional impairment	Ipsilesional limb functional performance deficits varied with both the damaged hemisphere and severity of contralesional arm impairment, with the most severe deficits expressed in LHD participants with severe contralesional impairment	Level II
McCrea et. al.; 2003 [260]	Patients affected by stroke (20; 60.9 ± 6.1 years); Healthy control (10; 61 ± 9 years)	Assess muscle strength and time-dependent properties of muscle contraction in persons affected with stroke	All parameters were impaired in the more affected arm, whereas peak torque and time to develop torque were impaired in the less affected arm	Level III
Metrot et. al.; 2013 [261]	Patients affected by stroke (12; 63.7 ± 9.7 years); No healthy control	Assess the natural evolution of reaching kinematics during standard poststroke rehabilitation, focusing on bimanual coordination	For the paretic limb, amount of movement was lower for bimanual reaching compared with unimanual reaching (p < 0.05). For bimanual reaching, movement kinematics were similar for both limbs (p = 0.589)	Level IV
Morris et. al.; 2012 [262]	Patients affected by stroke (56; 67.8 ± 13.1 years); Healthy control (50; 67.8 ± 9.9 years)	Investigate effects of bilateral training on ipsilesional arm dexterity and activity limitation, Explore the relationships between contralesional and ipsilesional recovery	The bilateral training group demonstrated significantly greater change in dexterity (P > 0.03) during the intervention phase. There was no significant correlation between ipsilesional and contralesional recovery	Level I
Noskin et.al.; 2008 [263]	Patients affected by stroke (30; 61.5 ± 11.3 years); No healthy control	Investigate ipsilateral hand performance after hemiparetic stroke, starting in the early post-stroke period	The initial degree of impairment of grip strength in the contralateral hand did not correlate with the degree of impairment of 9HPT in either the contralateral or ipsilateral hand (p = 0.98), whereas the initial degree of impairment of 9HPT in the contralateral hand correlated with the degree of impairment of 9HPT in the ipsilateral hand (p = 0.035)	Level IV
Olczak et. al.; 2022 [264]	Patients affected by stroke (60; 65.3 ± 14.43 years); No healthy control	Analyze the effect of the position of the trunk and the affected upper limb on the coordination and grip strength of the affected dominant and non-dominant hand and wrist in people after ischemic stroke	Higher and relevant results were observed in the non-dominant hand, in the supine position in terms of motor coordination parameters of the fingers and wrist, and grip strength (p < 0.05)	Level II
Patel et. al.; 2019 [265]	Patients affected by stroke (13; 68.04 ± 12.17 years); Healthy control (13; 69.79 ± 7.94 years)	Investigate the impact of stroke on dynamic bimanual force control and compare the contribution of each hand to a bimanual task	The proportion of force contributed by the non-paretic hand reduced and force variability of the non-paretic hand increased during the force decrement phase	Level IV
Pohl et. al.; 2000 [266]	Patients with stroke affecting their right (10; 71.7 ± 13.7 years) & left side (10; 67.2 ± 13.3 years) & (10; 68.7 ± 8.1 years); No healthy control	Investigate whether motor control deficits are present in the ipsilateral UE when there is mild impairment in the contralateral UE	The ipsilateral UE of just the stroke patients with left brain damage presented control deficits (p < 0.05)	Level III
Pollet et. al.; 2022 [267]	Patient affected by stroke with a low grip strength (10; 64.2 ± 15.9 years) and a high grip strength ≥ 45 (11; 68.4 ± 7.8 years); Healthy control (10; 67.1 ± 7.8 years)	Determine how grip strength capacity of the paretic hand influences its contribution to bimanual tasks	The amount of force contributed by the paretic hand increased in bimanual tasks with an increase in its grip strength capacity (p < 0.01). In the bimanual MVC task and bimanual force control task, both hands contributed equal magnitudes of force in the high strength-capacity group but unequal forces in low strength-capacity group	Level III
Prados-Roman et. al.; 2020 [268]	Patients affected by stroke (20; 56.95 ± 14.09 years); Healthy control (20; 57.1 ± 13.22 years)	Evaluate maximal handgrip strength, fatigue resistance, grip work, and muscle fatigue in mildly affected stroke persons	Persons with stroke demonstrated significantly reduced handgrip performance regarding maximal handgrip strength, resistance to fatigue, grip work, and muscle fatigue for the contralesional hand	Level III
Reale et. al.; 2021 [269]	Patients affected by stroke (20; 69.2 ± 10.1 years); No healthy control	(1) Analyze the correlation between the actigraphic index and the severity of 90-day disability as quantified by the mRS (2) Establish the ability of the AR24H index to predict 90-day disability preliminarily	The actigraphic AI positively correlates with the 90-day mRS (p < 0.001)	Level II
Rinehart et. al.; 2009 [270]	Patients with RHD (12; 59.2 ± 9.9 years) & LHD (17; 65.1 ± 11.9 years); Healthy control (25; 64 ± 8.3 years)	Determine if right hand preference influences the relative use of each limb after stroke	(1) Ipsilesional arm use was greater after RHD than LHD; (2) the LHD group used both arms together more often than the RHD group but less often than the control group; and (3) both stroke groups used their contralesional, paretic arm to the same degree	Level II
Rose et. al.; 2005 [271]	Patients affected by stroke (30; 63 years); Healthy control (30; 67 years)	Determine the role of anticipatory and movement control processes for the coordination of bimanual target aiming in individuals post stroke	The nonparetic limb exhibited a prolonged movement time in the bimanual condition. Compared with the unimanual condition, the nonparetic limb exhibited a lower peak velocity in the bimanual condition (p < 0.05)	Level III
Rose et. al.; 2013 [272]	Patients affected by stroke (30; 63.17 ± 12.17 years); 30 healthy controls (no demographics data)	Quantify interlimb coordination strength and compare individuals with asymmetric effector ability poststroke to nondisabled controls	Interlimb coupling was significantly stronger for the nondisabled compared to the stroke group	Level III
Siekierka-Kleiser et. al.; 2006 [273]	Patients affected by stroke (52; 62 ± 14 years); Healthy control (9; 60 ± 8 years)	Investigate whether the recovery dynamics of hemiparetic patients with motor hemineglect are different from those in hemiparetic patients without motor hemineglect	Patients without motor hemineglect showed an increase in spontaneous movement activity (p < 0.05), while patients with motor hemineglect showed no such increase (p > 0.1)	Level II
Soares et. al.; 2015 [274]	Patients affected by stroke (42; 61.7 ± 10 years); No healthy control	Verify if the functional hemispheric asymmetry plays an influence over the deficits of upper limbs of hemiparetic stroke patients, as well as if these possible alterations are different between men and women	The tests performed showed a difference between the paretic and non-paretic sides (p < 0.05). Hemispheric dominance was different in the paretic limb, with the best performance in BBT on the dominant side (p = 0.016)	Level II
Song et. al.; 2013 [275]	Patients affected by stroke (8; 45 ± 11 years); No healthy control	Investigate the parameters during voluntary arm tracking at different velocities for evaluating motor control performance after stroke	The affected and unaffected sides were different during the arm tracking experiment (P < 0.05)	Level IV
Toba et. al.; 2021 [276]	Patients affected by stroke (35; 59.16 ± 13.79 years); No healthy control	Introduce an objective and quantitative assessment method for MN, based on differential actigraphy which provides continuous assessment of spontaneous movements over 24 h	Differential actigraphy showed asymmetrical activity corresponding with the side of hemispheric brain damage suggesting unilateral elementary motor disorders, MN, or both disorders in these patients	Level II
Uswatte et. al.; 2000 [277]	Patients affected by stroke (9; 54.4 ± 19.5 years); Healthy control (12; 21 ± 2.5 years)	Evaluate whether transforming accelerometer recordings with a threshold filter provides an objective measure of the duration of arm movement when patients are outside the laboratory and cannot be directly observed by experimenters or clinicians	Correlations between the threshold-filtered recordings and the observer ratings of the duration of arm, torso, and ambulatory movements were 0.93, 0.93 and 0.99, respectively	Level IV
Uswatte et. al.; 2006 [278]	Patients affected by stroke (82; 63 ± 12.8 years); Healthy control (87; 64.2 ± 12.7 years)	Test whether the ratio of impaired-arm to unimpaired-arm accelerometer recordings converges with measures of real-world, impaired-arm use and diverges from a measure of overall physical activity	Correlations calculated across all participants at baseline between the ratio of more-impaired to less-impaired arm accelerometer recordings and AAUT and MAL scores were .60 and .52, respectively	Level I
Varghese et. al.; 2020 [279]	Patients affected by stroke (42; 59.16 ± 12.3 years); No healthy control	Investigate the relationship between the motor capacities of the two hands differs based on the side of stroke	Compared to RHD, the relationship between contralesional arm impairment and ipsilesional hand motor capacity was stronger (p = 0.03) in LHD	Level II
McCombe Waller et. al.; 2006 [280]	Patients affected by stroke (16; 59 ± 12.62 years); No healthy control	Examine the characteristics of bilateral simultaneous and bilateral sequential paretic-lead and nonparetic-lead functional reaching tasks at preferred and fast speeds	Despite hemiparesis, the two arms demonstrate a temporal coupling when moving simultaneously. At faster speeds, paretic arm's movement time was better when reaching with or before the non-paretic arm	Level IV
Whitford et. al.; 2018 [281]	Patients affected by stroke (8; 57.03 ± 6.64 years); No healthy control	Determine the effects of in-home high dose accelerometer-based feedback on (1) perception of paretic UE use; (2) actual paretic UE use; and (3) capability in individuals’ chronic post-stroke in the home setting	Participants had significant perceived gains in how much (p = 0.017) and how well (p = 0.050) they used the paretic UE. There were no significant group changes in actual paretic UE AOU or capability	Level II
Yang et. al.; 2021 [282]	Patients affected by stroke (14; 60.97 ± 11.9 years); Healthy control (14; 59.31 ± 10.58 years)	Measure functional grasp movements in individuals with chronic stroke and healthy controls throughout all activities over multiple days using a novel wrist-worn device	A larger AI (p = .01) of the affected hand in the stroke group were found compared to that of the nondominant hand in the control group	Level III

*3D* Three Dimensional, *AAUT* Actual amount of use test, *ADL* Activities of Daily Living, *AHA* Assisting Hand Assessment, *AI* Asymmetry Index, *AOU* Amount Of Use, *AR24H* Asymmetry Rate Index for 24 Hour, *ARAT* Action Reaction Arm Test, *BBT* Box and Block test, *BPI* Brachial Plexus Injury, *CP* Cerebral Palsy, *CST* Corticospinal Tract, *DBS* Deep Brain Stimulation, *EA* Error Augmentation, *EDSS* Expanded Disability Status Scale, *EMG* Electro Myography, *FMA* Fugl-Meyer Assessment, *HABIT* Hand Arm Intensive Bimanual Training, *HCP* Hemiparetic Cerebral Palsy, *LHD* Left Hemisphere Damage, *MACS* Manual Ability Classification System, *MAL* Motor Activity Log, *MCID* Minimal Clinically Important Difference, *MMG* Mechanomyography, *MN* Motor Neglect, *mRS* modified Rankin Scale, *MS* Multiple Sclerosis, *MWF* Myelin Water Fraction, *NHPT* Nine-Hole Peg Test, *NIHSS* National Institute of Health Stroke Score, *PD* Parkinson's Disease, *PMT* Peg Moving Task, *PNI* Peripheral Nerve Injury, *RHD* Right Hemisphere Damage, *SCI* Spinal Cord Injury, *SCZ* Schizophrenia, *SD* Standard Deviation, *UE* Upper Extremity, *USCP* Unilateral Spastic Cerebral Palsy, *VR* Virtual Reality

**Fig. 2:**
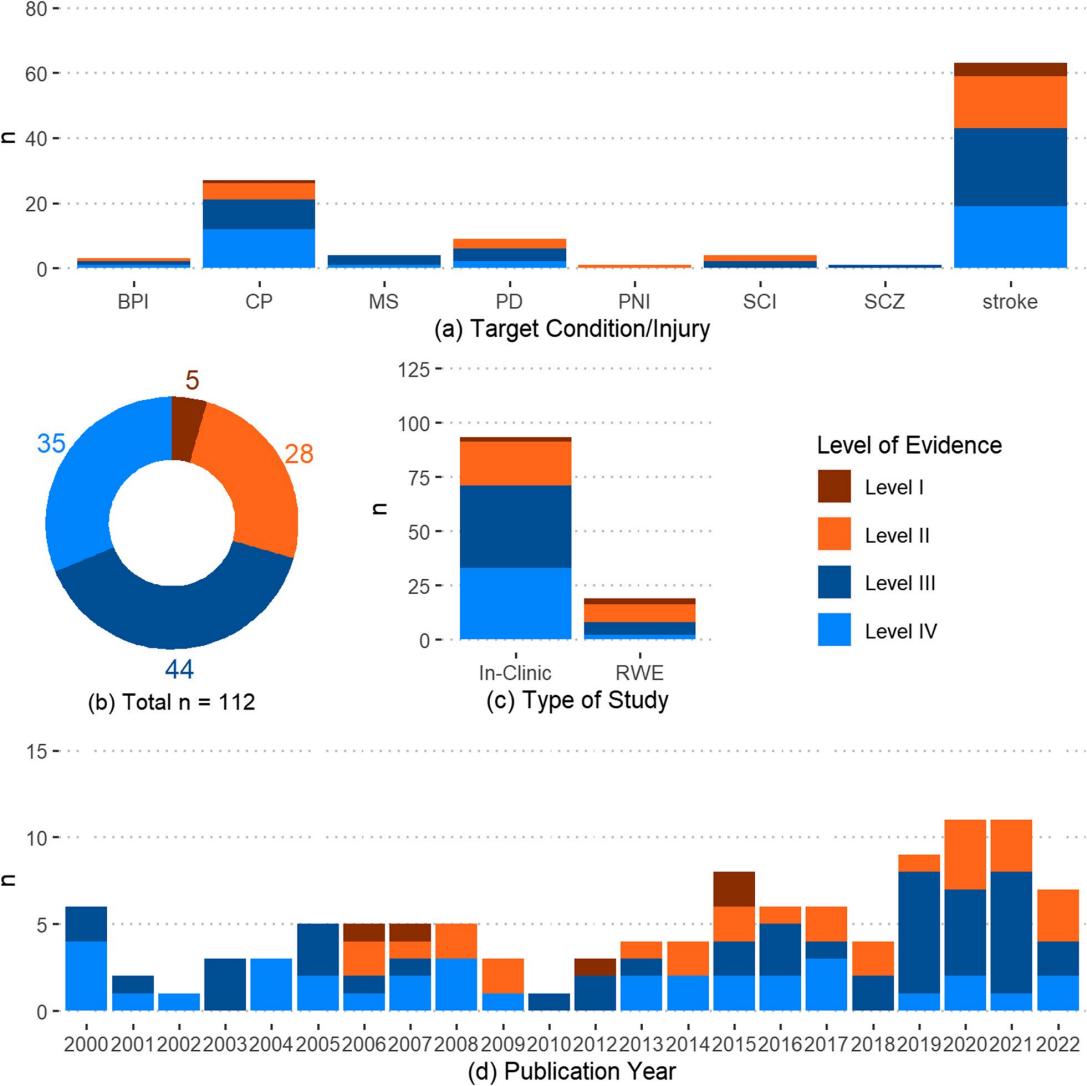
Number of articles divided by** a** target condition/injury,** b** level of evidence,** c** type of study, and **d** publication year (n = number of articles)

The selected articles concentrated on BPI, CP, MS, PD, PNI, SCI, Schizophrenia (SCZ), and stroke. It is important to note that even though SCZ is not a nervous system lesion or injury, it is a neurological disorder and presents with upper extremity behavioral asymmetry. Stroke was the most prominent condition (63 articles) followed by CP (27 articles). PNI, and SCZ were the least studied conditions/injuries (1 article each) (Fig. 2a).

### Critical appraisal of sources

The reviewed articles had a higher proportion of Level III and IV of evidence (44 and 35 counts respectively). Five articles were at Level I evidence, and 28 articles contained Level II evidence (Fig. 2b and Table 2). The number of Level II and III articles increased over the years (Fig. 2d), with the increase being concentrated over 2018 to 2021. 93 articles performed data collection in a clinical setting, while 19 articles collected complete or partial RWE (Fig. 2c). An upward trend was seen in the number of articles published over the years, with the highest number of articles published in 2020 and 2021 (11 articles each) (Fig. 2d).

### Extracted data

**Fig. 3:**
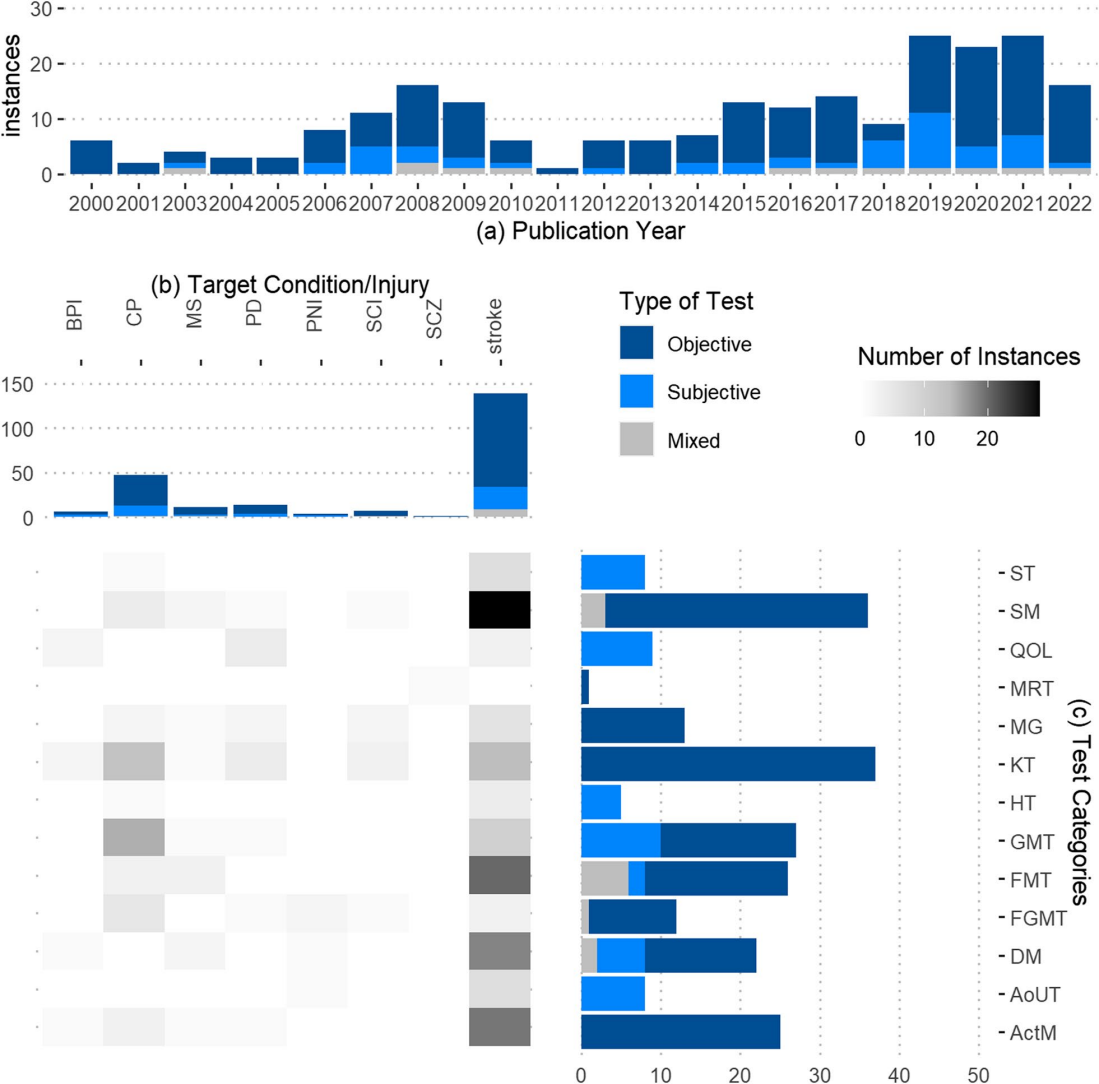
Heat map distribution of the tests performed in the selected articles by target condition/injury and test categories; Number of articles divided by** a** publication year,** b** target condition/injury,** c** test categories

The tests and sensors used in the selected studies were divided into thirteen categories based on the type of test and data collected (Table 3). SGB divided the tests into respective categories based on each test’s description (as stated in databases such as the Shirley Ryan Ability Lab rehabilitation measure database) and data collected. Kinematics tests were the most common types of tests performed (37 instances) followed by strength measures (36 instances). Motion reflex test was the least common type of test used (1 instance) (Fig. 3c). The articles contained 153 instances of objective tests, 48 instances of subjective tests, and 11 instances of mixed data type tests (Table 3). Objective data collection increased between 2000 to 2021 (from 6 to 18instances) (Fig. 3a). Stroke had the highest number of objective tests (105 instances) and subjective tests (25 instances), followed by CP (34 and 12 instances, respectively) (Fig. 3b). All activity monitoring, electromyography, kinematic, and motion reflex tests collected objective data, while amount of use, handedness, quality of life, and spasticity tests collected subjective data. Some disability tests of fine motor skills or strength collected mixed data. Strength measures were the most popular outcome measures for stroke (28 instances) followed by fine motor tests (20 instances), activity monitoring (19 instances) and disability measures (18 instances). Gross motor tests were used primarily on the CP treatment groups (15 instances).

**Table 3:** Categories of tests and sensors used in the selected articles

Categories	Tests and sensors used in the articles	Instances	Type of test	Articles
Activity Monitoring (ActM)	Triaxial Accelerometry	14	Objective	[174, 175, 180, 201, 213, 222, 223, 229, 240, 250, 254, 269, 273, 276, 281]
	Uniaxial Accelerometry	9	Objective	[179, 233, 234, 238, 251, 252, 270, 277, 278]
	Upper Limb Activity Monitor (Accelerometry)	1	Objective	[233]
Amount of Use Tests (AoUT)	Actual amount of use test	1	Subjective	[278]
	Motor Activity Log	7	Subjective	[214, 232, 237, 251, 254, 278, 281]
Disability Measures (DM)	Arm Motor Ability Test	1	Mixed	[230, 270]
	Disabilities of Arm, Shoulder, and Hand	2	Subjective	[174, 214]
	Expanded Disability Status Scale	2	Subjective	[202, 203]
	Fugl-Meyer Assessment	14	Objective	[230, 234, 237, 242, 249, 250, 254, 258, 259, 265, 267, 268, 271, 279]
	Functional Independence Test	1	Subjective	[252]
	modified Rankin Scale	1	Subjective	[269]
Fine and Gross Motor Tests (FGMT)	Bruininks–Oseretsky Test of Motor Proficiency	1	Mixed	[179]
	custom reaching task	3	Objective	[191, 205, 214]
	Distractor test	1	Objective	[227]
	Jebsen–Taylor Test of Hand Function	6	Objective	[177, 179, 186, 214, 218, 259]
Fine Motor Tests (FMT)	Action Reaction Arm Test	6	Objective	[222, 223, 248, 251, 252, 262]
	Computerized Peg Moving Test	1	Objective	[176]
	Aiming-Tapping Task	1	Objective	[183]
	Melbourne Assessment	2	Subjective	[180, 238]
	Nine-Hole Peg Test	9	Objective	[201, 203, 204, 221, 232, 240, 262, 263, 274]
	Grooved Pegboard Test	1	Objective	[259]
	Wolf Motor Function Test	6	Mixed	[249–252, 279, 281]
Gross Motor Tests (GMT)	Assisting Hand Assessment	7	Subjective	[175, 177–179, 181, 184, 238]
	Box and Block test	9	Objective	[194, 201, 221, 224, 232, 238, 241, 242, 274]
	Caregiver Functional Use Survey	1	Subjective	[179]
	Custom coordination tests	8	Objective	[181, 182, 187, 189, 199, 255, 261]
	Manual Ability Classification System	2	Subjective	[175, 184]
Handedness Tests (HT)	Edinburgh handedness inventory	5	Subjective	[175, 231, 234, 265, 267]
Kinematics Tests (KT)	Electrogoniometer	6	Objective	[190, 204, 212, 233, 236, 275]
	Electromagnetic Motion Tracking	10	Objective	[173, 187, 195, 228, 247, 248, 263, 273, 274, 282
	Inertial Measurement Unit	4	Objective	[173, 187, 210, 232]
	Optical Motion Capture	17	Objective	[18, 178, 182, 186, 188, 192, 196–198, 206, 209, 216–218, 235, 264, 266]
Motion Reflex Test (MRT)	modified Traffic Light paradigm	1	Objective	[219]
Myography (MG)	Electromyography	11	Objective	[186, 200, 202, 213, 217, 218, 230, 231, 239, 275, 282]
	Mechanomyography	2	Objective	[211, 239]
	36 item Short Form survey	1	Subjective	[174]
Quality of Life Surveys (QOL)	World Health Organization Quality of Life	1	Subjective	[174]
	Stroke Impact Scale	3	Subjective	[254, 278, 281]
	Unified Parkinson's Disease Rating Scale	4	Subjective	[208] [210] [211] [212]
Spasticity Tests (ST)	Modified Ashworth Scale	8	Subjective	[190, 230, 237, 249, 258, 265, 271, 274]
Strength Measures (SM)	Force Transducer based tests	33	Objective	[186, 193–195, 202, 207, 220, 221, 236, 237, 239, 242–244, 247, 249, 251–253, 256–260, 263–265, 267, 268, 274, 282]
	Graded redefined assessment of strength, sensibility and prehension	1	Mixed	[215]
	Motricity Index	2	Mixed	[202, 276]

**Fig. 4:**
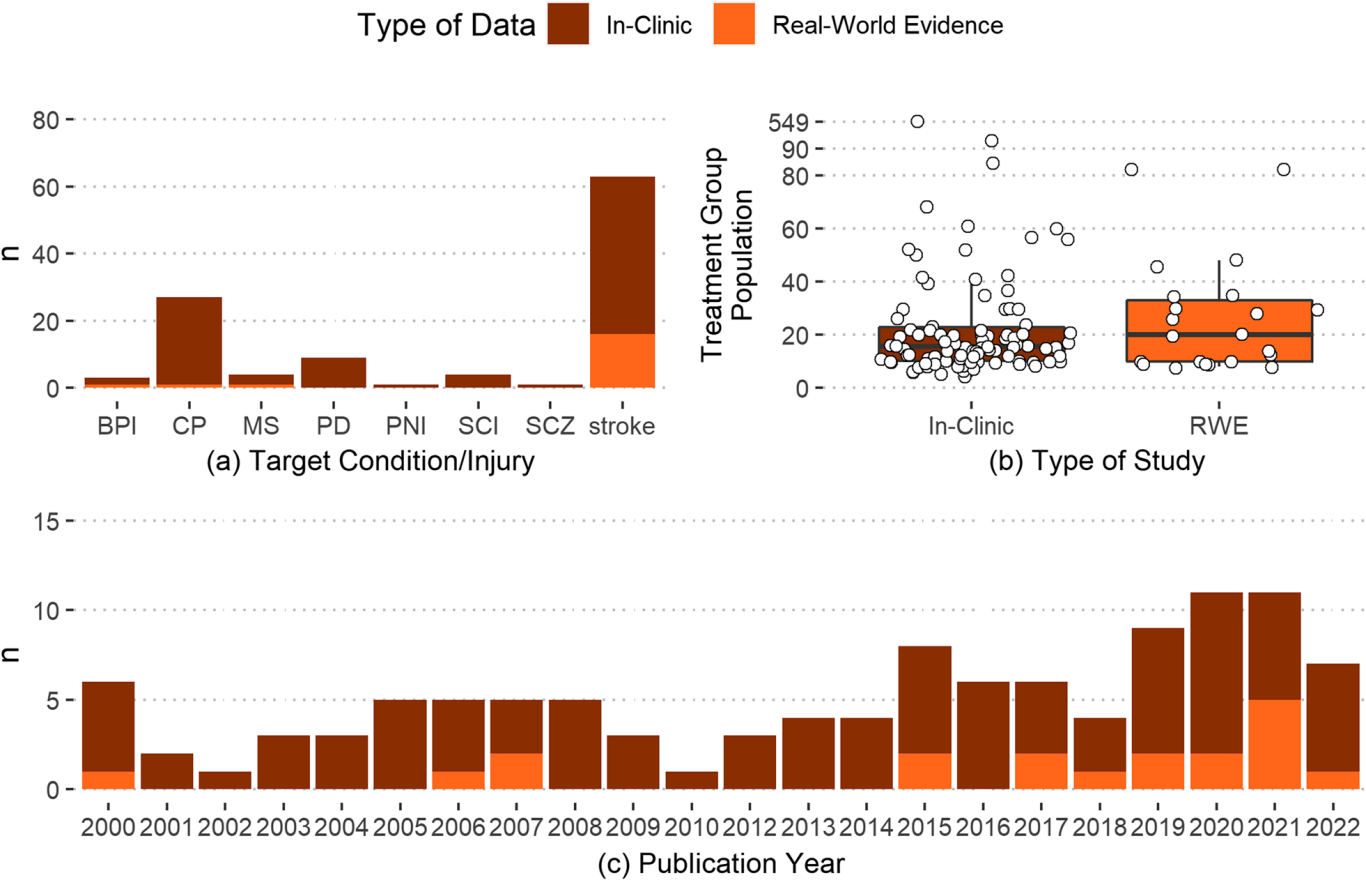
Distribution of the data collection method for** a** target condition/injury, **b** treatment group population vs. type of study, and** c** publication year (n = number of articles)

Most of the articles reviewed performed tests and collected data in the clinic (93 articles). Articles on stroke as the target condition saw most of the real-world data collection (16 articles) (Fig. 4a). Studies using RWE based tests had a higher treatment group population (median: 20) compared to studies using in-clinic tests (median: 16) (Fig. 4b). The number of studies utilizing real-world data increased between 2000 and 2021 with only 1 out of 6 articles in 2000 compared to 5 out of 11 articles in 2021 containing some form of RWE (Fig. 4c).

## Discussion

### Summary of evidence

The evolution of the science for assessing UE asymmetry secondary to neurological conditions/injuries from 2000 to 2022 was reviewed. Most articles were Level III or IV of evidence. Stroke and CP were the most studied conditions. Tests collecting data on the kinematics and strength were widely used. RWE based testing and objective data collection increased between 2000 and 2021.

Stroke and CP were the most common conditions studied. Although CP and the other neurological conditions affect the patient chronically, stroke resulted in the most deaths, while also causing chronic paralysis in the patients who survived. Research funding allocated to stroke by the National Institute of Health (NIH) has seen a rise of 51% between 2008 and 2021, $296 million to $447 million respectively [283]. Within the same period, funding for CP increased by 7% ($28 to $30 million), PD by 67% ($152 to $254 million), SCI by 26% ($80 to $101 million). The funding for MS and SCZ reduced by 25% ($169 to $126 million) and 2% ($249 to $242 million) respectively [283]. No data was found for BPI and PNI. The neurological conditions other than stroke and PD are underfunded and under-studied. Hence, it is recommended that future research endeavors focus on a more diverse patient population affected by neurological conditions/injuries.

A higher proportion of the studies reviewed were case-controlled or poor cohort/ case-controlled studies (LoE Level III and IV). The quality of a study is largely dependent on its design. It is widely accepted that RCTs (LoE Level I) are the “gold standard”, but there are certain disadvantages associated with them (e.g., expensive to conduct, monitoring biases, quality is dependent on degree of randomization, etc.) [284]. Due to these disadvantages, cohort studies (LoE Level II) are often preferred. Case–control studies are retrospective (LoE Level III), hence do not explore the incidence of the outcome. Case–control studies also have many shortcomings as discussed by Lewallen et. al. [285]. Hence, future studies on UE asymmetry should be designed as either a RCT or a cohort study (LoE Levels I or II).

Most of the tests performed in the articles collected objective data. Qualitative tests often require less personnel training to collect, are less time consuming, and do not require expensive equipment or sensors. However, they often depend on a skilled observer or a dedicated patient to report the outcome based on a set guideline. The subjective data collected is dependent on perception or feeling at the time of the test. They also require skilled observers and if not attentive, they may inadvertently induce bias. Objective data provides a more unambiguous and unbiased representation of outcome. The use of objective data for quantifying UE asymmetry increased between 2000 and 2022, and further studies are necessary to determine their applicability and reproducibility.

The reviewed articles extensively employed tests to capture the kinematics of patients across all studied conditions and injuries. Among the various methods used for studying the subject's kinematics, Optical Motion Capture (OMC) stood out as the most prominent. According to an article that reviewed the state of the art in human motion tracking, Optoelectronic measurement systems (also known as OMC systems) were found to be more accurate than other systems, but they relied on proper calibration [286]. In contrast, Electromagnetic motion tracking systems were less accurate than OMC. They exhibited greater susceptibility to electromagnetic noise and had a lower sampling rate [286]. On the other hand, Inertial Measurement Units (IMUs) gained significance as a wireless and marker less motion tracking technology in recent years. IMU-based systems offered advantages such as being lightweight, cost-effective, and portable. However, there were certain concerns related to angle calculation that needs careful consideration [287]. Therefore, selecting the appropriate motion tracking system became crucial and depended on the specific use-case at hand.

Force transducers, primarily handheld dynamometers, were the dominant type of test used, appearing in 33 instances. As Mendoza et al. highlighted, handheld dynamometry (HHD) offered an efficient, objective, sensitive, and cost-effective method for quantifying strength [288]. Nevertheless, recent literature indicates that HHD is prone to intertester variability, meaning that different testers may produce varying results when using the same device [289]. Additionally, the reliability of HHD is influenced by the strength of the tester, particularly when assessing larger muscle groups. This aspect must be considered while interpreting the results obtained through HHD measurements.

Activity monitoring tests were one of the popular forms of outcome measure applied. Hollis et. al. supported the use of accelerometry by stating that it “…is not contaminated by learning and practice effects that may occur with repeated administration of standard measures.” [238]. When used to measure limb use asymmetry, accelerometry has been well correlated with standard clinical assessments [174, 290]. De Lucena et. al. stated “If quality of movement during daily life is an outcome important to people with a stroke, perhaps kinematic analysis of accelerometry provides a window to assess it.” [232]. Lakhani et. al. suggested the use of accelerometry in conjunction with other outcome measures to predict impairment in individuals with chronic stroke [250]. Toba et. al. emphasized the importance of the method used to analyze the actigraphy data [276]. Hence, when used appropriately, activity monitors have the potential to become a valid outcome measure in clinical practice.

Studies adopting RWE based outcome measures have increased in the recent years. Rehabilitation of patients with neurological conditions/injuries aims at improving the use of the affected UE in daily living tasks [233]. Webber et. al. noted that “…collection of real world data places minimal burden on subjects and provides quantitative arm usage information previously inaccessible to clinicians.” [174]. Similar observations were made by other articles [240, 252, 278]. The U.S. Food and Drug Administration (FDA) emphasized the importance of real world data and real world evidence to supplement clinical data in medical device clearance and best practices development [291]. RWE based approaches provide a better understanding of the patient’s condition in their day-to-day life as opposed to data collection in a research/clinical setting [35]. Pau et. al. pointed out the shortcoming of in-clinic tests, stating “…clinical tests capture only limited information about individuals’ actual upper limb dysfunction…” [201]. John Doyle stated “Real-world patients are fundamentally different than clinical trial patients.”, hence supporting the use of RWE in medical device testing [292]. All these testimonies recommend RWE and data be used in prognostic, diagnostic, and rehabilitative care of patients. There are number of limitations associated with RWE as stated by Kim et. al. (need for experts for data analysis, high possibility of bias, lack of standardization, etc.) [293]. RWE based tests often require an extended period of data collection. These limitations make it difficult to maintain a large subject size. All studies reviewed had a statistically significant result but might have been underpowered since a power analysis was not provided. Hence, it is recommended that a power analysis be performed for RWE based studies and the population size be large enough to ensure reliable statistical analyses.

### Limitations

There are limitations inherent to retrospective, scoping reviews. Only articles published within the range of 2000 and 2022 were considered for the review. Articles were selected from a list of articles only published in English (including English language translations). Hence, there is a possibility of missing the knowledge from publications in other languages. The software used for identifying the duplicate articles in the master list was trusted and the results were not cross referenced. There is a chance that some articles could have been overlooked due to the terms used for the electronic search. Hence, the search involved using multiple synonyms to reduce the risk of data loss. This review focused on multiple conditions and injuries. The quality assessment tools used for these conditions/injuries, though similar, still have some differences. We used the LoE to judge all the articles to facilitate a fair comparison. Systematic reviews, case studies, and book chapters were excluded from our review.

### Conclusions

The discussed limitations notwithstanding, this review demonstrated the following:

- More randomized controlled trials or cohort studies (LoE I or II) are needed in studies on UE asymmetry to improve the level of evidence being reported.
- Real-world outcome measures should be collected more frequently.
- Objective outcome measures should be given more importance.
- UE asymmetry for neurological conditions other than stroke need to be studied.
- Adequate power analysis must be performed to ensure reliable analyses.

## Data Availability

The datasets used and/or analyzed during the current study are available from the corresponding author on reasonable request.

## Abbreviations

3D: Three dimensional
AAUT: Actual amount of use test
ActM: Activity monitoring
ADL: Activities of daily living
AHA: Assisting hand assessment
AI: Asymmetry index
ALS: Amyotrophic lateral sclerosis
AoU: Amount of use
AoUT: Amount of use tests
AR2_24: Asymmetry rate index for the 24-hour period
ARAT: Action reaction arm test
BBT: Box and block test
BPI: Brachial plexus injury
CP: Cerebral palsy
CST: Corticospinal tract
DBS: Deep brain stimulation
DM: Disability measures
EA: Error augmentation
EDSS: Expanded disability status scale
EMG: Electro myography
FGMT: Fine and gross motor tests
FMA: Fugl-Meyer assessment
FMT: Fine motor tests
GMT: Gross motor tests
HABIT: Hand–arm bimanual intensive therapy
HCP: Hemiparetic cerebral palsy
HT: Handedness tests
KT: Kinematics tests
LHD: Left hemisphere damage
LoE: Sackett’s level of evidence
MACS: Manual ability classification system
MAL: Motor activity log
MCID: Minimal clinically important difference
MN: Motor neglect
mRS: Modified Rankin scale
MRT: Motion reflex test
MS: Multiple sclerosis
MWF: Myelin water fraction
NHPT: Nine-hole peg test
NIHSS: National Institute of Health Stroke Scale
PD: Parkinson's disease
PMT: Peg moving task
PNI: Peripheral nerve injury
QOL: Quality of life surveys
RCT: Randomized controlled trial
RHD: Right hemisphere damage
RWE: Real world evidence
SCI: Spinal cord injury
SCZ: Schizophrenia
SD: Standard deviation
SM: Strength measures
ST: Spasticity tests
UE: Upper extremity
USCP: Unilateral spastic cerebral palsy
VR: Virtual reality
